# Circulating MicroRNAs as Potential Predictors of Anthracycline-Induced Troponin Elevation in Breast Cancer Patients: Diverging Effects of Doxorubicin and Epirubicin

**DOI:** 10.3390/jcm9051418

**Published:** 2020-05-11

**Authors:** Sonia Gioffré, Mattia Chiesa, Daniela Maria Cardinale, Veronica Ricci, Chiara Vavassori, Carlo Maria Cipolla, Serge Masson, Maria Teresa Sandri, Michela Salvatici, Fabio Ciceri, Roberto Latini, Lidia Irene Staszewsky, Giulio Pompilio, Gualtiero I. Colombo, Yuri D’Alessandra

**Affiliations:** 1 Immunology and Functional Genomics Unit, Centro Cardiologico Monzino-IRCCS, 20138 Milan, Italy; s.gioffre91@libero.it (S.G.); mchiesa@ccfm.it (M.C.); vricci@ccfm.it (V.R.); cvavassori@ccfm.it (C.V.); gcolombo@ccfm.it (G.I.C.); 2 Cardiology Division, European Institute of Oncology, IRCCS, 20141 Milan, Italy; daniela.cardinale@ieo.it (D.M.C.); carlo.cipolla@ieo.it (C.M.C.); 3 Dipartimento di Medicina Clinica e Chirurgia, Università degli Studi di Napoli Federico II, 80138 Napoli, Italy; 4 Dipartimento di Scienze Cliniche e di Comunità, Università degli Studi di Milano, 20122 Milan, Italy; gpompilio@ccfm.it; 5 Istituto di Ricerche Farmacologiche Mario Negri IRCCS, 20156 Milan, Italy; serge.masson@roche.com (S.M.); roberto.latini@marionegri.it (R.L.); lidia.staszewsky@marionegri.it (L.I.S.); 6 Laboratory Medicine Division, Humanitas Clinical and Research Center, IRCCS, 20089 Milan, Italy; maria.sandri@humanitas.it (M.T.S.); michela.salvatici@humanitas.it (M.S.); 7 Hematology/Transplant Unit, Istituto Scientifico H. San Raffaele IRCCS, 20132 Milan, Italy; ciceri.fabio@hsr.it; 8 Vascular Biology and Regenerative Medicine Unit, Centro Cardiologico Monzino IRCCS, 20138 Milan, Italy

**Keywords:** microRNA, anthracyclines, biomarkers

## Abstract

Anthracyclines are anti-neoplastic drugs presenting cardiotoxicity as a side effect. Cardiac troponins (cTn) and echocardiography are currently used to assess cardiac damage and dysfunction, but early biomarkers identifying patients in need of preventive treatments remain a partially met need. Circulating microRNAs (miRNAs) represent good candidates, so we investigated their possible roles as predictors of troponin elevation upon anthracycline treatment. Eighty-eight female breast cancer patients administered with doxorubicin (DOX) or epirubicin (EPI) were divided into four groups basing on drug type and cTn positive (cTn+) or negative (cTn−) levels: DOX cTn−, DOX cTn+, EPI cTn− and EPI cTn+. Blood was collected at baseline, during treatment, and at follow-up. We identified plasma miRNAs of interest by OpenArray screening and single assay validation. Our results showed miR-122-5p, miR-499a-5p and miR-885-5p dysregulation in DOX patients at T0, identifying a signature separating, with good accuracy, DOX cTn− from DOX cTn+. No miRNAs showed differential expression in EPI subjects. Conversely, an anthracycline-mediated modulation (regardless of cTn) was observed for miR-34a-5p, -122-5p and -885-5p. Our study indicates specific circulating miRNAs as possible prediction markers for cardiac troponin perturbation upon anthracycline treatment. Indeed, our findings hint at the possible future use of plasma miRNAs to predict the cardiac responsiveness of patients to different anticancer agents.

## 1. Introduction

Over the past three decades, the breakthroughs in breast cancer treatment strategies—including the addition of new generation systemic agents, as well as the use of more advanced and precise radiotherapy techniques—have significantly improved patient survival [1]. However, anthracyclines—chemotherapeutic agents isolated from *Streptomyces peucetius* bacteria introduced into clinical oncology in the 1960s [2]—still represent the cornerstone of treatment in breast cancer and in many other solid tumors. Anthracyclines inhibit DNA and RNA synthesis by intercalating between the base pairs and by inhibiting the activity of topoisomerase II [3]. Among this family, doxorubicin (DOX) and epirubicin (EPI) are the most widely used medications in breast cancer. The two drugs are structurally similar, with EPI differing from DOX in the epimerization of the 4-hydroxyl group of the amino sugar moiety. Both are metabolized in the liver and eliminated through the bile [4]. Despite the clinical benefits, treatment with anthracyclines is hampered by cardiotoxicity, a side effect ranging from subclinical ventricular dysfunction to severe cardiomyopathy and heart failure, possibly leading to cardiac transplantation or death [5]. The toxicity profiles of the two molecules are different since at equal doses, EPI seems to be less toxic than DOX [4]. Since anthracycline-induced cardiotoxicity may be identified at different times during or after drug treatment, it can be classified into three types: (i) acute, when occurring after a single dose or not later than two weeks from the end of treatment; (ii) early-onset chronic, developing within one year; and (iii) late-onset chronic, developing years after the end of treatment. However, recent data suggest that cardiotoxicity is a continuum that starts with myocardial cell injury, followed by progressive cardiac dysfunction, which leads to overt heart failure if disregarded and not treated [6,7]. The cytotoxic effect of anthracyclines is based on DNA intercalation, but the exact mechanisms leading to anthracycline-induced cardiotoxicity remain unclear. Several hypotheses have been proposed such as DNA damage, oxidative stress, alterations in lipid metabolism, and mitochondrial membrane depolarization leading to cardiomyocyte death [8].

The assessment of anthracycline-associated cardiotoxicity is usually conducted by echocardiography, monitoring cardiac function following chemotherapy administration [9]. In a position paper, the Task Force for cancer treatments and cardiovascular toxicity of the European Society of Cardiology defined cardiotoxicity as a decrease below the lower limit of the normal left ventricular ejection fraction (LVEF) in echocardiography, set to 50% [10]. The LVEF-based diagnosis, though, is not useful for the early assessment or for prevention, because cardiotoxicity is often a late event and echocardiography detects significant functional impairments only after their occurrence. On the other hand, a preserved LVEF after treatment does not exclude future cardiac deterioration.

To date, the most commonly used circulating markers indicating the possible onset of anthracycline-induced cardiotoxicity are cardiac troponins I and/or T (cTnI or cTnT) [11]. Cardiac troponins are released upon heart damage and are considered the golden standard as biomarkers of myocardial injury since their detection in plasma is indicative of cardiomyocyte necrosis [12]. Cardiac troponins are currently used to help clinicians in establishing the cardiac-monitoring schedule of anthracycline-treated patients [13], although their use is not yet recommended as routine by the guidelines. Indeed, in the absence of the perturbation of cTn levels (cTn−), patients are classified as low-risk, thus not requiring strict and prolonged observation of heart function. Conversely, cTn-positive (cTn+) patients have a greater incidence of major adverse cardiac events, and the persistence of cTn elevation is an indicator of greater cardiac impairment and a higher incidence of future events [14,15], even if this does not necessarily result in symptomatic heart failure or cardiac death. Indeed, cardiac troponins are considered very good negative biomarkers but have a sub-optimal positive predictive potential.

For all these reasons, there is a need to find novel, specific and accurate biomarkers of cardiotoxicity onset that allow early assessment and monitoring using minimally invasive approaches.

In recent years, several investigations have focused on the possible involvement of microRNAs (miRNAs) in DOX-induced toxicity. MiRNAs are small noncoding, endogenous, single-stranded RNAs that regulate gene expression by either inhibiting the translation of messenger RNAs (mRNA) or by promoting their degradation [16]. They play a key role in many biological processes, under both physiological and pathological conditions. Increasing evidence shows that miRNAs are involved in all cardiac functions, including the conductance of electrical signals, heart muscle contraction, and growth. Interestingly, circulating miRNAs have been proposed as noninvasive and specific markers of several cardiovascular diseases [17,18,19,20]. They are demonstrated to be highly stable over time in various body fluids, including plasma, serum, urine and saliva [21], and their levels can be assessed by sensitive techniques such as quantitative real-time PCR (RT-qPCR) [22].

Articles assessing circulating miRNAs’ potential as biomarkers of DOX-induced cardiotoxicity in breast cancer patients are still very limited, showing significant heterogeneity in terms of cardiotoxicity assessment, the number of enrolled patients, and the specific treatment [23,24]. Moreover, only a limited number of miRNAs have been examined. Furthermore, a few authors have investigated circulating miRNA as a predictor of resistance to EPI in breast cancer patients.

Here, we investigated plasma miRNA expression in breast cancer patients before they underwent either DOX or EPI administration, with the aim of showing their potential in helping clinicians in the selection of the least cardiac-harmful treatment for the patients and/or in identifying high-risk patients to be treated with cardio-preventive treatment before chemotherapy.

## 2. Materials and Methods

### 2.1. Patients

The patients included in this study were recruited from the ICOS-ONE (International CardiOncology Society-one) clinical trial [25]. In particular, 88 female breast cancer patients treated with either DOX (*n* = 32) or EPI (*n* = 56) were included in this study. Patients with a history or clinical evidence of heart failure and/or ischemic heart disease, a previous history of chemotherapy or showing increased levels of cardiac troponin at baseline were excluded. The trial complied with the Declaration of Helsinki. All patients gave written informed consent. The protocol was approved by the Regulatory Agencies and IEO Ethics Committee (approval code IEO S701/412, Milan, Italy) and was registered in the ClinicalTrials.gov registry (NCT01968200) before starting (EudraCT Number: 2012-002248-26). The cumulative anthracycline dose was calculated by converting the different anthracycline doses in terms of doxorubicin equivalents [26]. Further information about the inclusion and exclusion criteria can be found in [25].

### 2.2. Cardiac Troponins

Since troponins were assayed in local laboratories with commercial reagents measuring different subunits (I or T) with different sensitivities, troponin levels were normalized to the local upper limits of normal (ULN) and expressed as a binary variable, higher (>, cTn+) or lower (≤, cTn−) than the local ULN. All data about troponins are presented in Table S1. Troponin was assessed at baseline, at each anthracycline cycle and 1, 3, 6 and 12 months after the completion of chemotherapy (CT).

### 2.3. Cardiac Function Evaluation

The cardiac function of all subjects was assessed by echocardiography, and 2D-echo was conducted in at least three consecutive cycles from the parasternal long-axis and mitral and papillary level short-axes and apical 4- and 2-chamber views. Left ventricular volumes were calculated and normalized to body surface area, and the modified biplane Simpson’s rule was applied to calculate LVEF.

### 2.4. Total RNA Isolation and MicroRNA Retrotranscription

Blood samples were collected into tubes containing EDTA and were immediately centrifuged at 3000 rpm for 10 min to separate the plasma, and stored at −80 °C until further use. Total RNAs were isolated from 100 µL of plasma using the Total RNA Purification Plus kit (Norgen, Thorold, ON, Canada) following the manufacturer’s instructions for liquid samples. RNA pellets were resuspended in 50 µL of RNAse free water and stored at −80 °C.

### 2.5. Circulating MicroRNA Screening

The screening was performed on a selected cardiovascular disease-associated miRNA panel using an OpenArray Real-time PCR System (Thermo Fisher Scientific, Waltham, MA, USA), according to the manufacturer’s protocol for liquid samples. In particular, we used custom plates allowing the detection of 112 miRNAs (Table S2). Starting from 2 µL of total RNA, miRNA retrotranscription was conducted using TaqMan Advanced miRNA cDNA synthesis (Life Technologies, Waltham, MA, USA), according to the manufacturer’s protocol. A mixture composed of 2.5 µL of each amplified cDNA and 2.5 µL of TaqMan OpenArray Real-Time PCR master mix (Applied Biosystems, Waltham, MA, USA) was dispensed on an OpenArray plate using the AccuFill automated sample loading system (Applied Biosystems, Waltham, MA, USA) and run using a QuantStudio 12k Flex Real-Time PCR System (Thermo Fisher Scientific, Waltham, MA, USA). Data analysis was conducted using cloud-based Thermo Fisher Scientific proprietary software (Thermo Fisher cloud, https://apps.thermofisher.com/apps/). Samples presenting a Ct value >35, an amplification score (Amp Score) <1.24 and a Ct confidence value <0.8 were excluded. The expression data for each sample are presented in Table S3. Normalization was conducted using the Global Mean Normalization method implemented by the software.

### 2.6. Single MicroRNA Assay Validation

All miRNAs, showing a fold change >2 and <0.6 with a *p*-value < 0.05, were selected for validation using single TaqMan Advanced miRNA assays (Life Technologies, Waltham, MA, USA), according to manufacturer’s protocol. In these experiments, miR-16 was used as the normalizer because of its strong and stable expression, mirroring results from Global Mean Normalization in the screening step as previously described [18,20,27].

### 2.7. Statistical Analysis

In order to determine the expression levels of plasma miRNAs, raw Ct values were processed with the formula 2^–delta-delta Ct (2^−ΔΔCt^), using miR-16 as the reference miRNA, and the geometric mean of Ct for each miRNA in the control subjects as the calibrator sample [28]. Finally, the data were log-transformed and expressed as –ΔΔct, to obtain a normally distributed dataset. The miRNA relative expression, as well as the demographic variables, were expressed as mean ± standard deviation. Boxplots and connected scatter plots were drawn by GraphPad Prism (v 5.0, GraphPad Software, La Jolla, CA, USA). Differential expression analyses were performed by linear model tests, using R’s native functions (https://www.r-project.org/). *p*-values < 0.05 were deemed statistically significant. The logistic regression classifier was implemented by the “caret” package [29] while a 3D scatterplot and a receiver operating characteristic (ROC) curve were plotted by the “rgl” (v 0.100) and the “ROCR” (v 1.0.7) [30] packages, respectively.

## 3. Results

### 3.1. Study Population

**Figure 1:**
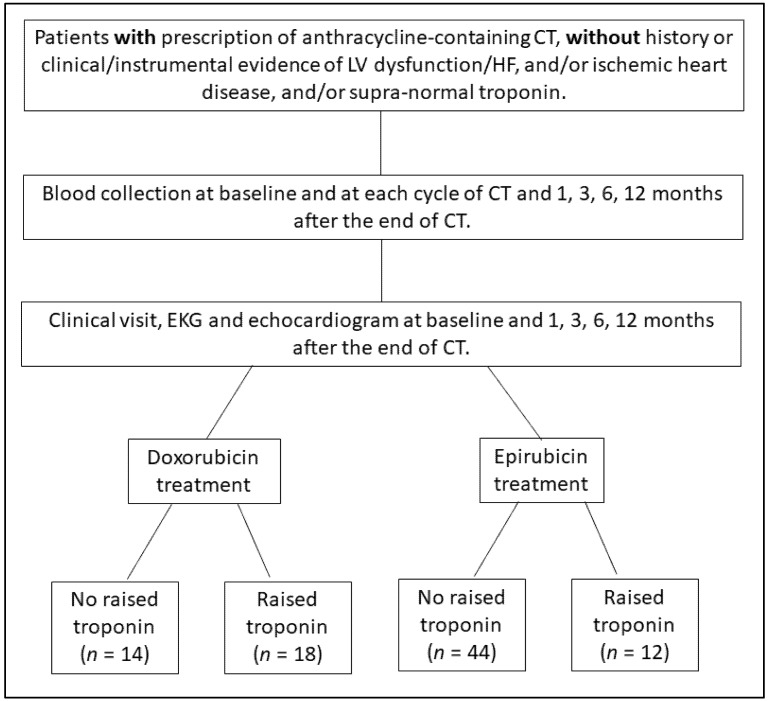
Flow chart of the study. CT: chemotherapy; LV, left ventricle; HF, heart failure; EKG: electrocardiogram.

**Figure 2:**
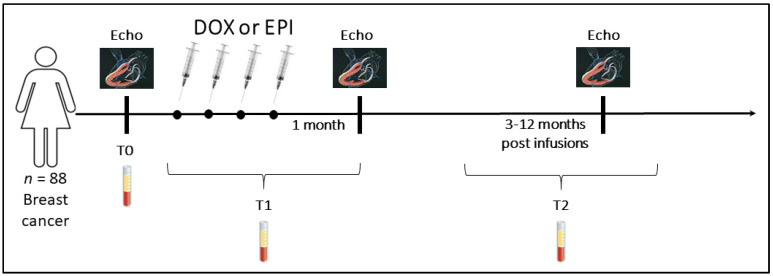
Experimental design. Eighty-eight breast cancer patients were enrolled and treated with doxorubicin (DOX) or epirubicin (EPI). Thick black vertical bars indicate experimental time-points when echocardiography was performed and blood samples collected.

Based on the troponin plasma levels evaluated during and after treatment, patients were divided into four groups (Figure 1): DOX-treated patients without elevation of troponins (DOX cTn−, *n* = 14); DOX-treated with elevation of troponins (DOX cTn+, *n* = 18); EPI-treated without elevation of troponins (EPI cTn−, *n* = 44); and EPI-treated with elevation of troponins (EPI cTn+, *n* = 12). Demographic and clinical data are summarized in Table 1. We considered three time points: baseline (before treatment, T0); T1 (during treatment, up to one month after the last infusion); and T2 (long-term follow-up, from three to twelve months after the last infusion) (Figure 2). None of the evaluated patients showed signs of cardiac dysfunction at the 12-month follow-up. Indeed, the mean LVEF showed no appreciable variation from baseline values in any group, as reported in Table 1.

### 3.2. Plasmatic MicroRNA Profiling

Since the main purpose of the present study was to identify circulating miRNAs potentially associated with a future troponin increase upon anthracycline treatment, we conducted an array-based screening on samples collected at baseline (i.e., before treatment). We compared cTn+ vs. cTn− in both the DOX and EPI groups (*n* = 12/group, demographic data in Table S4). The results showed five miRNAs as potentially regulated (*p* < 0.05) in the DOX group and four in the EPI group. In particular, miR-99b-5p, -122-5p, -125b-5p, -532-5p and -885-5p presented a putative upregulation in the DOX cTn+ vs. cTn− group, while miR-128-3p, -181b-5p, -181c-5p and -361-3p were downregulated in EPI cTn+ vs. cTn− patients (Table S5).

### 3.3. Differential Expression of Plasma MicroRNAs at Baseline

**Figure 3:**
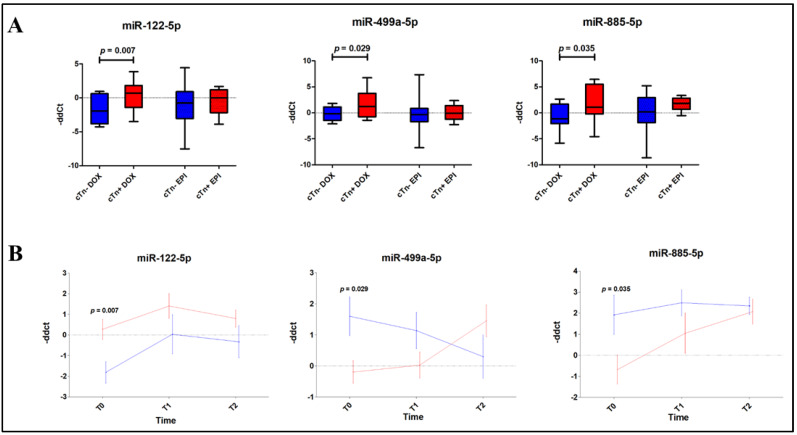
Differential expression of DOX plasma miRNAs. (**A**) Three miRNAs, miR-122-5p, -499a-5p and -885-5p, showed modulated expression in DOX cTn+ vs. DOX cTn− patients at baseline. Data are depicted as box-and-whisker plots and expressed as max, median and min. (*n* = 14 DOX cTn−; *n* = 18 DOX cTn+; *n* = 44 EPI cTn−; *n* = 12 EPI cTn+). (**B**) Time course of miR-122-5p, -499a-5p and -885-5p, showing modulated expression in cTn+ vs. cTn− DOX patients at baseline but not at T1 and T2. Vertical bars represent the mean of −ΔΔCT ± SEM. Blue, cTn− DOX; Red, cTn+ DOX. (*n* = 14 DOX cTn−; *n* = 18 DOX cTn+). DOX, doxorubicin; miRNAs, microRNAs; EPI, epirubicin; cTn−, negative troponin levels; cTn+, positive troponin levels; −ΔΔCT, –delta-delta Ct; SEM, standard error of the mean.

Following the screening step, we analyzed the expression of the putatively regulated miRNAs in all T0 samples by single assays. Moreover, miR-1-3p, miR-34a-5p and miR-499a-5p were evaluated based on their known involvement in cardiac diseases and DOX-induced cardiotoxicity [24]. Our results showed that miR-122-5p (*p* = 0.007), miR-499a-5p (*p* = 0.029) and miR-885-5p (*p* = 0.035) presented a higher expression in DOX cTn+ vs. in DOX cTn− patients (Figure 3A and Table S6). These differences were not observed in EPI subjects and no difference in miR-1-3p or miR-34a-5p expression was observed in either treatment group. Conversely, we did not confirm any significant modulation of the miRNAs detected in the screening phase in the EPI group.

### 3.4. Doxorubicin Treatment Modulates Circulating MicroRNA Expression

The three miRNAs differentially expressed at baseline were then also evaluated at the following time points (T1 and T2) in DOX-treated patients, comparing cTn+ vs. cTn− subjects. Interestingly, miR-122-5p showed a trend of higher expression, despite the differences not reaching statistical significance at T1 and T2. However, both miR-499a-5p and -885-5p presented overlapping levels of expression at these time points (Figure 3B and Table S7).

### 3.5. A Circulating MicroRNA Signature at Baseline is Associated with Increased cTn after DOX Treatment

**Figure 4:**
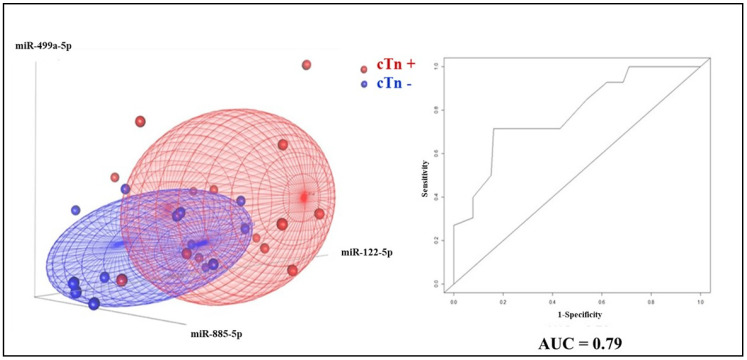
Baseline plasma miRNA-based identification of DOX-cTn+ and DOX-cTn− patients. 3D scatterplots (left) and ROC analyses (right) were used to investigate whether the DOX-related plasma miRNA expression at baseline could be used to correctly separate cTn+ from cTn− patients. The AUC assesses the performance of the logistic model built, taking into account miR-499a-5p, miR-885-5p and miR-122-5p. Blue: cTn−. Red: cTn+. AUC: area under the curve.

These results prompted us to investigate whether the expression of the three miRNAs presenting differential levels of expression at baseline in DOX-treated patients could be used to predict increased cardiac troponin before treatment. To this aim, we performed a binary logistic regression analysis using the means of the expression levels of these three miRNAs at baseline as the independent variable and found that they areauc associated with future increases in cTn; indeed, as shown by the ROC curve analysis in Figure 4, the model exhibits an Area Under the Curve (AUC) of 0.79. Notably, 3D scatterplot analysis showed good performance of this miRNA signature in separating DOX-cTn+ and DOX-cTn− patients.

### 3.6. Circulating MicroRNA Modulation upon Anthracycline Treatment

**Figure 5:**
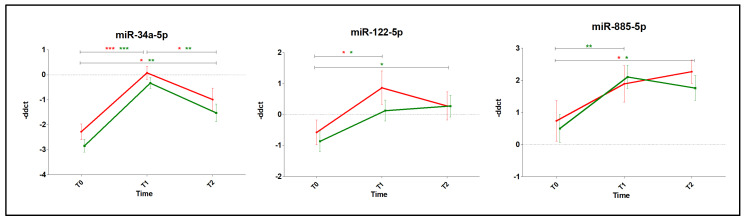
Circulating miRNAs modulated upon anthracycline treatment. Time course for miR-34a-5p, -122-5p and -885-5p showing modulated expression in DOX (red) and EPI (green) patients. Vertical bars represent mean –ΔΔCT ± SEM. Green, DOX; Orange, EPI. * *p* < 0.05; ** *p* < 0.01; *** *p* < 0.001.

We finally analyzed the effects of the two different drugs (DOX and EPI) on the expression of plasma miRNAs regardless of troponin levels. Our data showed heterogeneous regulation for the three miRNAs (miR-34a-5p, -122-5p and -885-5p) over time. Interestingly, miR-34a-5p presented a significant anthracycline-induced upregulation at every time point. Conversely, miR-122-5p presented an upregulation only at T1 (vs. T0) in the DOX group and at T1 and T2 (vs. T0) in the EPI group. MiR-885-5p was shown to be upregulated by both drugs at T2 vs. T0 and also at T1 vs. T0 by EPI (Figure 5 and Table S8).

## 4. Discussion

This is the first study comparing circulating miRNA expression in plasma of breast cancer patients upon treatment with two different components of the anthracycline family, DOX and EPI. Our results clearly indicate that, despite their structural similarity, the two drugs do not affect circulating miRNAs in the same way in breast cancer patients. This seems to hint to a different mechanism of action leading to a differential response to each specific treatment. Most importantly, we have identified a signature composed of three miRNAs, miR-122-5p, miR-499-5p, and miR-885-5p, that was able to predict, with good accuracy, adverse cardiac responses to DOX before its administration. In order to illustrate that, we focused our attention on the main biomarker of cardiac damage, cTn; indeed, if increased during and/or after anthracycline treatment, cTn represents an indicator of the possible onset of heart dysfunction [31]. Of note, cTn is known to possess very good negative predictive value, i.e., the absence of cTn elevation upon treatment excludes future dysfunction onset. Conversely, an increase in cTn upon drug administration can indicate an increased propensity to develop cardiac dysfunction, but without providing any information about the timing of the possible onset [14].

Two recent studies investigated circulating miRNAs in breast cancer patients as possible predictors of cardiotoxicity upon treatment with either EPI or DOX. In the first case, Qin et al. showed the regulation of eight miRNAs in the plasma of breast cancer patients who were treated with EPI and suffered from cardiac dysfunction, but none of them were similarly regulated in the present work. This is probably due to different experimental settings and different detection and normalization methods. In particular, the authors selected 14 miRNAs to be evaluated, based on their previously proposed pro-angiogenic role, and arbitrarily used U6, a non-miRNA small-ncRNA, as a normalizer; this makes comparison with our results very difficult [32]. In the case of the DOX-focused study, Todorova and co-authors investigated the differences in miRNA plasma expression at baseline and after the first administration cycle between patients with normal and patients with decreased LVEF after chemotherapy completion. They identified several miRNAs as being differentially expressed in the two groups, but none of them showed any overlap with our findings. Again, this is possibly due to the different experimental approaches and miRNA evaluation techniques. In particular, the LNA-based screening method adopted by this group differs greatly from our TaqMan-based approach in terms of accuracy, sensitivity and the number of miRNAs evaluated. More importantly, their experimental design was very different to ours, because the authors investigated miRNA expression changes between baseline and the first cycle of DOX-based chemotherapy. Interestingly, though, miR-34a-5p showed a DOX-induced upregulation in both groups, regardless of LVEF perturbation, a result in line with our findings [23].

Other previous studies investigating DOX-mediated cardiotoxicity focused their attention on miRNAs showing a perturbed expression upon anthracycline treatment. Rigaud and collaborators identified circulating miR-1 as a potential marker of cardiotoxicity in breast cancer patients, showing its regulation by DOX treatment. Interestingly, none of the miRNAs evaluated in the manuscript (including miR-1) showed any difference at baseline when comparing patients with and without later cardiotoxicity presentation [33]. However, Leger and co-authors investigated the effects of DOX in young cancer patients, showing that plasma miR-29b and -499 are acutely elevated after anthracycline therapy [34]. This latter result fits well with our data, although we have no specific information about miRNA expression in the acute phase of the treatment.

Our work presents some limitations. First, the sample size of our cohorts is small. This lessens the generalizability of the work but paves the way to new studies in which pre-determining a differential and harmful response to an antineoplastic treatment could be used to help clinicians in their choice of the most effective and safe therapeutic route. Of note, none of the evaluated patients developed cardiac dysfunction, as evidenced by the lack of decrease in the LVEF. This result could be seen as a limitation of the study, as it prevents the correlation and evaluation of miRNA expression at baseline as a real marker of functional anthracycline-induced impairment. Furthermore, we failed to identify miRNAs showing potential in predicting EPI-induced cTn increases; this may be possibly due to lower cardiac toxicity or to different mechanisms by which this drug induces heart damage/dysfunction. Indeed, while it is known that epirubicin is less cardiotoxic than doxorubicin [35], very few studies have been conducted to investigate the exact mechanisms of this discrepancy in terms of detrimental cardiac effects [36], which thus remain quite elusive. Another explanation could be the restricted number of miRNAs evaluated during the screening phase. Thus, we cannot exclude that other miRNAs could be exploited as predictors of EPI-induced increases in cardiac troponin, an issue that could be addressed by using “wide-coverage” screening methods like microarrays and/or next-generation sequencing. Another limitation regards troponin assessment. Since these patients were derived from a multi-center study, they were enrolled and evaluated in different hospitals, using different assays, with either high or normal sensitivity. To partially overcome this issue, we transformed the troponin level into a dummy variable for patient stratification. A final limitation is the absence of patients with clear left ventricular dysfunction among our cases. Therefore, the full clinical relevance of our findings should be further investigated in future studies.

**Figure 6:**
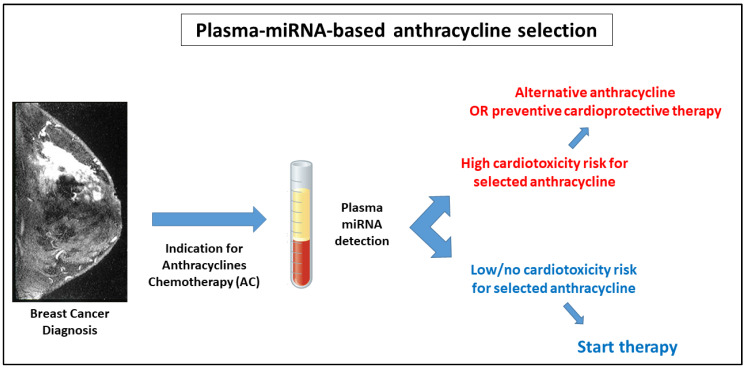
Circulating miRNAs as Anthracyclines Chemotherapy decision-makers. Flow chart of the possible use of plasma miRNA assessment at baseline to help clinicians in selecting drugs with the lowest cardiotoxicity risk and/or deciding whether to start cardioprotective preventive therapies.

Despite these limitations, our work can be regarded as proof of the concept that the circulating miRNA signature could be used to ascertain the patient’s cardiac responsiveness to different classes of anthracycline. These observations may pave the way to the identification of early biomarkers to help clinicians in the choice of chemotherapeutic drugs with maximal efficacy and minimal risk of cardiac toxicity for each individual patient or, alternatively, in adopting preventive cardioprotective treatments (Figure 6). Indeed, the identification of predictive biomarkers is of utmost importance, particularly for childhood cancer survivors. Previous investigations have shown that the treatment and outcome data from large cohorts of patients help in assessing individual risks for subsequent heart failure with reasonable accuracy [37]. Our work falls perfectly in line with these kinds of studies, aiming at the identification of clinically useful models that allow the prediction of cardiovascular risk before the actual treatment begins.

## 5. Conclusions

Unlike previous investigations, our work aimed to show a different approach to the prevention of anthracycline-induced heart damage. Indeed, we showed that circulating miRNAs could be a key for future personalized and safe anticancer-drug selection, thus reducing the number of patients in need of additional cardioprotective treatments.

## Figures and Tables

**Table 1:** Demographic and clinical characteristics.

	EPI cTn−	EPI cTn+	*p*-Value	DOX cTn−	DOX cTn+	*p*-Value
Subjects (*n*)	44	12		14	18	
Age (mean ± SD)	49.3 ± 11.4	52.2 ± 6.3	0.31	53.9 ± 10.8	53.3 ± 11.3	0.41
BMI (mean ± SD)	23.3 ± 3.2	25.4 ± 3.2	0.08	26.2 ± 3.8	25.3 ± 3.3	0.4
Smokers, *n* (%)	5 (11)	3 (25)	0.19	4 (29)	5 (28)	1
Hypertension, *n* (%)	2 (4)	0 (0)	1	0 (0)	0 (0)	1
Dyslipidemia, *n* (%)	3 (7)	1 (8)	1	3 (21)	1 (0)	0.25
Diabetes, *n* (%)	0 (0)	1 (8)	0.2	0 (0)	0 (0)	1
Cycles of therapy (mean ± SD)	3.6 ± 0.5	3.7 ± 0.7	0.9	4.0 ± 0	4.0 ± 0	1
Anthracycline cumulative dose * mg/m^2^ (mean ± SD)	223.3 ± 42.5	239.1 ± 45.3	0.26	240 ± 0	226.2 ± 38.7	0.43
LVEF (%) at T0 (mean ± SD)	63.2 ± 4.2	64.4 ± 6.9	0.77	64.3 ± 4.2	65.4 ± 7.1	0.94
LVEF (%) at 1 year (mean ± SD)	62.4 ± 5.5	63.9 ± 3.5	0.56	63.6 ± 6.3	66 ± 5.9	0.43

SD, standard deviation; EPI, epirubicin; DOX, doxorubicin; cTn−, negative Troponin levels; cTn+, positive troponin levels; BMI: Body Mass Index; LVEF: Left Ventricular Ejection Fraction. * Cumulative anthracycline dose was calculated by converting the different anthracycline doses in terms of doxorubicin equivalents [10,26].

## Supplementary Materials

The following are available online at https://www.mdpi.com/2077-0383/9/5/1418/s1, Table S1: Troponin data of all evaluated patients, Table S2: miRNA detection probes on OpenArray Arrays, Table S3: miRNA Screening Expression Values, Table S4: Screening Patients Demographics, Table S5: Plasma miRNAs Screening Results, Table S6: Normalized log-transformed expression values for significant miRNAs at baseline, Table S7: DOX miRNAs Expression Values, Table S8: Changes over time of relevant miRNAs in DOX and EPI whole groups.
